# Trauma Care Systems in Urban Areas: Our Thoughts on the Contrasting Approaches in South Korea and Japan

**DOI:** 10.7759/cureus.53737

**Published:** 2024-02-06

**Authors:** Hiroyuki Otsuka, Ichiro Okada, Kiyohiko Adachi, Michihiro Takeda, Toshiki Sato

**Affiliations:** 1 Emergency and Critical Care Medicine, Tokai University Hachioji Hospital, Tokyo, JPN; 2 Critical Care Medicine and Trauma, National Hospital Organization Disaster Medical Center, Tokyo, JPN; 3 Emergency and Critical Care Medicine, Tokai University School of Medicine, Kanagawa, JPN

**Keywords:** centralized, urban areas, trauma surgery, trauma center, inclusive trauma care system

## Abstract

Trauma is a major global health issue, causing significant mortality, disability, and healthcare expenses. Since 2012, the Ajou Trauma Center in South Korea has been at the forefront, providing centralized severe trauma care for a population of 9.5 million. In 2022, the center managed 3,500 cases of severe trauma, including 500 helicopter transports, and conducted 2,800 surgeries, with 450 addressing torso trauma. Its exceptional performance has garnered global recognition, solidifying its position among the top advanced trauma centers.

In Tokyo, critically ill and major trauma patients are currently transported to the nearest emergency and critical care centers, each serving a population of approximately 0.5 to 0.6 million people. Due to the low incidence of trauma per facility and an aging population, implementing a high level of trauma care and a comprehensive training framework within Japan's existing system poses significant challenges.

A comparative analysis of South Korea's centralized system and Tokyo's decentralized approach indicates that the centralized system may lead to the establishment of a more advanced trauma center with ethical and equity considerations, compared to the decentralized approach. Therefore, consolidating major trauma cases in Tokyo shows promise for establishing exceptional trauma centers. This emphasizes the urgent need for Japan to take immediate steps towards a more robust future in trauma care. This assertion aligns with the global discourse on improving trauma care practices and could make a valuable contribution to the scholarly literature on trauma care systems.

## Editorial

Trauma is a leading cause of mortality, disability, and healthcare expenses worldwide [1]. In 2012, the South Korean government implemented the Regional Trauma Center system to address these issues. The system provides prompt and appropriate medical care to trauma patients by dividing the country into five regions and establishing 17 trauma centers [2]. In November 2023, we visited Professor Jung K. at the Ajou Trauma Center to gain insight into the current status of the system. This report compares the current findings in the urban area of South Korea with those in Japan.

The Ajou University Hospital Trauma Center serves as the Gyeonggi-do South Regional Trauma Center, catering to a population of 9.5 million in southern Gyeonggi-do. The trauma center has specialized facilities, including 100 beds dedicated to trauma care (40 ICU beds and 60 general ward beds), two resuscitation units, one CT room, one angiography room, and three operating rooms. The center employs 26 specialists, including 16 surgeons, who provide round-the-clock care. In addition, there are 251 specialized trauma care nurses, eight trauma coordinators, and other personnel to enhance trauma care services. The center is also planning further upgrades.

In 2022, the Ajou Trauma Center admitted around 3,500 patients with severe trauma, including 1,300 major trauma patients with an Injury Severity Score (ISS) of over 15 and 500 helicopter cases. Approximately 2,800 surgeries have been performed at this center, with roughly 450 involving torso trauma. These numbers are exceptional compared to other medical centers in Japan. Helicopter operations are available 24/7 and are vital for patient transportation. The Doctor Helicopter system operates during the day, while at night, patients are only accepted from fire and police services. The ratio of patients accepted is approximately seven to three during the day and night, respectively. Additionally, the Ajou Trauma Center is registered with the American College of Surgeons Trauma Quality Improvement Program, demonstrating a commitment to improving trauma care quality. It achieved results comparable to those of top-level trauma centers in advanced countries [3]. In summary, the centralized system enables the establishment of a high-capacity trauma center that is at the forefront of the field.

On the other hand, in Tokyo, major trauma patients are transported to the nearest emergency and critical care center, similar to all critically ill patients. Each center serves a population of around 0.5 to 0.6 million people. Japan's reputation as a highly safe country results in a low incidence of both blunt and penetrating trauma, with a low proportion of severe trauma cases in overall emergency medical care due to an aging population [4].

A multicenter study was conducted in the Tama region of Tokyo in 2022 and presented at the 2023 Annual Meeting of the Japan Surgical Society [5]. Eight emergency and critical care centers serve a population of 4.2 million in the Tama region of Tokyo. The study found that approximately 1.4% of emergency patients transported to the emergency and critical care centers had suffered from severe trauma and were hemodynamically compromised. The frequency of cases per facility is generally low, with an average of only a few cases per month. Surgical procedures for trauma cases are rare at each facility, especially for hemodynamically unstable torso trauma. These surgeries are performed only once a month, if at all (Table 1). The establishment of a trauma center relies heavily on having a sufficient number of severe cases. Establishing an efficient trauma care and training framework in Tokyo poses a challenge due to the rarity of such cases per facility. However, Tokyo's large population makes it feasible to establish a prominent trauma center that can provide appropriate medical care and training by consolidating severe trauma patients.

**Table 1:** The average number of patients transported to each facility per month The data has been represented as Mean±SD.

Variable	
Trauma patients with hemodynamic instability	2.8 ± 2.4
Trauma patients who underwent surgery	8.1 ± 5.0
Patients who underwent craniotomy	0.7 ± 0.4
Patients who underwent torso-trauma surgery	1.5 ± 0.9
Patients who underwent orthopedic or plastic surgery	4.9 ± 3.0
Patients who underwent interventional radiology	1.0 ± 0.6

A meticulous comparative analysis between South Korea's centralized trauma care system and Tokyo's decentralized approach underscores the pressing need for Japan to promptly initiate comprehensive measures towards fortifying the future of trauma care. This imperative not only resonates with the ongoing global discourse on the advancement of trauma care practices but also holds the potential to make a substantial contribution to the scholarly literature surrounding trauma care systems.

South Korea's centralized model has demonstrated efficacy and efficiency in delivering trauma care, providing a noteworthy benchmark. In stark contrast, Tokyo's decentralized approach reveals the current challenge of each facility handling a limited number of severe trauma cases. The conclusion drawn from this comparative analysis suggests that the centralized system may lead to the establishment of a more advanced trauma center, taking into account ethical and equity considerations compared to the decentralized approach. Tokyo's potential consolidation of major trauma cases with administration including the usage of helicopters could pave the way for the establishment of exceptional trauma care centers.

In light of these findings, it becomes paramount for Japan to expedite strategic initiatives. This is especially crucial given the dynamic landscape of advanced trauma care practices globally, aligning with the imperative to elevate healthcare standards universally. The outcome of Japan's swift action not only promises valuable insights into the country's trauma care system but also has the potential to significantly enrich the scholarly discourse surrounding trauma care systems on a global scale.

The potential impact of centralizing trauma care in Japan can bring about several significant effects. Specifically, the specific impacts that this approach may have include: 1) specialized expertise: centralizing trauma care allows for the establishment of specialized trauma care centers, bringing together knowledge and expertise. This enables the development of highly skilled medical teams capable of delivering prompt and efficient trauma care; 2) streamlined and standardized protocols: the centralized system promotes consistency in treatment protocols. Specialized trauma care centers can establish standardized procedures, ensuring the consistent application of best practices; 3) enhanced training and professional development: centralization facilitates continuous training and professional development for healthcare professionals. Concentrating severe trauma cases in specialized centers provides an environment where physicians can accumulate expertise, share knowledge, and engage in ongoing training programs; 4) optimized resource utilization: the centralized approach enables the efficient use of resources. Specialized trauma care centers can benefit from economies of scale, optimizing resource allocation, and promoting research collaborations; 5) establishment of advanced trauma centers: the comparative analysis suggests that a centralized system may lead to the establishment of more advanced trauma centers. This is particularly important in addressing ethical and equity considerations, ensuring that high-quality trauma care is accessible to a broader population; 6) potential for helicopter usage and administration: Tokyo's potential consolidation of major trauma cases, including the use of helicopters for transportation, could further enhance the speed and efficiency of trauma care delivery.

In summary, the centralization of trauma care in Japan has the potential to positively impact the country's healthcare system by fostering specialized expertise, standardizing protocols, promoting continuous professional development, optimizing resource utilization, and potentially establishing more advanced trauma centers. These outcomes not only elevate the quality of trauma care within Japan but also position the country to contribute significantly to the global discourse on trauma care systems.
